# Spontaneous and Microbiota‐Driven Degradation of Anthocyanins in an In Vitro Human Colon Model

**DOI:** 10.1002/mnfr.202300036

**Published:** 2023-07-31

**Authors:** Emad Shehata, Priscilla Day‐Walsh, Lee Kellingray, Arjan Narbad, Paul A. Kroon

**Affiliations:** ^1^ Quadram Institute Bioscience Norwich Research Park Norwich NR4 7UQ UK; ^2^ Chemistry of Flavour and Aroma Department National Research Centre 33 El Buhouth St. Dokki Cairo 12622 Egypt; ^3^ Department of Obstetrics and Gynaecology, University of Cambridge, The Rosie Hospital Robinson Way Cambridge CB2 0SW UK; ^4^ Centre for Trophoblast Research (CTR), Department of Physiology, Development and Neuroscience University of Cambridge Cambridge CB2 3EG UK

**Keywords:** anaerobic fermentation, bioavailability, colon model, flavonoids, gut microbiota, microbial metabolism, polyphenols

## Abstract

**Scope:**

The consumption of dietary anthocyanins is associated with various health benefits. However, anthocyanins are poorly bioavailable, and most ingested anthocyanins will enter the colon where they are degraded to small phenolic metabolites that are the main absorbed forms. Little is known about the processes of anthocyanin degradation in the gut and the role of the human gut microbiota. This study aims to determine the contribution of spontaneous and microbiota‐dependent degradation of anthocyanins in the human colon.

**Methods and results:**

Purified anthocyanin extracts from black rice and bilberry were incubated in an in vitro human fecal‐inoculated pH‐controlled colon model over 24 h and anthocyanins were analyzed using HPLC‐DAD. The study shows that the loss of anthocyanins occurs both spontaneously and as a consequence of metabolism by the gut microbiota. The study observes that there is high variability in spontaneous degradation but only modest variation in total degradation, which included the microbiota‐dependent component. The degradation rate of anthocyanins is also shown to be dependent on the B‐ring substitution pattern and the type of sugar moiety, both for spontaneous and microbiota‐dependent degradation.

**Conclusion:**

Anthocyanins are completely degraded in a model of the human colon by a combination of spontaneous and microbiota‐dependent processes.

## Graphical abstract

The vast bulk of absorbed metabolites of anthocyanins are degradation products that are formed in the colon, but how they are formed is not known. The study shows that degradation of anthocyanins occurs via both spontaneous (chemical) and gut microbiota‐dependent processes and that there is variation both within and between individuals.

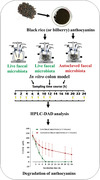

## 1 Introduction

**Figure 1:**
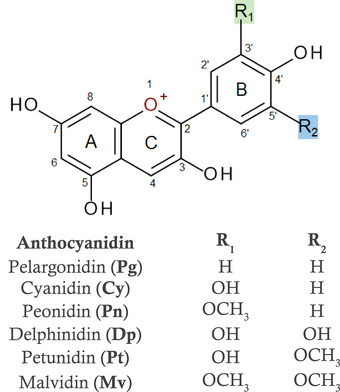
The basic structure and numbering system of anthocyanidins (anthocyanin aglycone).

Anthocyanins are polyphenols belonging to a subgroup of flavonoids and are the major water‐soluble pigments found in the plant kingdom, particularly the red, blue, and purple colors.^[^ ^1^ ^]^ Chemically, anthocyanins are an aglycone moiety (anthocyanidin) attached to sugar moiety via a glycosidic bond. Anthocyanidins differ in the number of hydroxy and/or methoxy substitutions in the B‐ring, giving six main anthocyanidins that are found in nature, for example, pelargonidin (Pg), cyanidin (Cy), peonidin (Pn), delphinidin (Dp), petunidin (Pt), and malvidin (Mv) (**Figure** **1**). Naturally occurring anthocyanins are commonly glycosylated in position 3 and position 5, typically as mono‐ and/or di‐glycosides.^[^ ^2^ ^]^ Plant foods such as blackcurrants, eggplant, bilberries, strawberries, and black rice are rich in anthocyanins.^[^ ^3^ ^]^ However, the content and composition of anthocyanins in plants vary considerably, for example, black rice predominantly contains cyanidin‐3‐*O*‐glycoside (Cy3Glc), while bilberry contains a mixture of at least 14 anthocyanins.^[^ ^4^, ^5^ ^]^

Evidence from epidemiological^[^ ^6^, ^7^ ^]^ and dietary intervention studies^[^ ^8^, ^9^, ^10^, ^11^, ^12^ ^]^ supports the notion that consuming anthocyanin‐rich diets and foods has beneficial effects on humans, particularly for cardiometabolic health. Data from numerous pre‐clinical studies provide evidence that they can have antioxidant, anti‐inflammatory, anti‐cancer, and antimicrobial activities.^[^ ^6^ ^]^ However, anthocyanin bioavailability has been shown to be very low, with urinary excretion accounting for <0.1% to a maximum of 2% of the ingested dose.^[^ ^13^ ^]^ Further, absorption of intact anthocyanins occurs early after ingestion (0.5–1.5 h),^[^ ^14^ ^]^ and this means that most anthocyanins pass through the small intestine and reach the colon. This suggests the colon is the most important site of anthocyanin degradation, as has been suggested by others.^[^ ^15^, ^16^ ^]^ Indeed, there is very clear evidence to support this in the report of De Ferrars et al.^[^ ^14^ ^]^ where it was shown that a small proportion of ring‐fission products from [5‐^13^C] Cy3Glc were absorbed in the first 1.5 h (i.e., from the small intestine), but the vast majority were absorbed over the next 24–48 h (i.e., from the colon).

There are limited reports of in vitro studies regarding the colonic metabolism of anthocyanins in humans. A few studies have investigated anthocyanin metabolism using models of the human colon, either with pH control^[^ ^17^ ^]^ or without pH control.^[^ ^18^, ^19^ ^]^ Some have reported the use of single bacterial strains isolated from human fecal samples to investigate anthocyanin degradation which is a simplification of the complex multi‐organism structures present in the human gut^[^ ^20^ ^]^ but is useful for determining the degrading capabilities of individual strains. Most other reported studies are concerned with degradation of anthocyanins in the absence of gut microbes, and while these might be useful in suggesting that spontaneous degradation is involved, they ignore the possibility of a role for the gut microbiota and are often done in conditions that are not like those found in the human colon, for example, at room temperature and in the presence of oxygen and light. Currently, important questions remain unresolved. For example, is the degradation of anthocyanins in the colon a spontaneous chemical degradation process, a microbiota‐dependent active process, or a combination of both? How variable is the degradation rate (*k*_deg_) of anthocyanins (and by definition the rate of production of metabolites) between fecal inoculum obtained from different stool donors, and between stools from the same donor? How do structural differences between different anthocyanins affect the rate of degradation?

The present work aimed to investigate the colonic metabolism of black rice and bilberry anthocyanins by the human gut microbiota using a pH‐controlled batch colon fermentation model aiming to i) determine the contribution of both spontaneous and microbiota‐driven processes, ii) assess the inter‐ and intra‐individual variation, and iii) study the effects of B‐ring substitution patterns, as well as glycosylation on the colonic metabolism of anthocyanins.

## 2 Results

All fecal samples that were used in the studies reported here have been given a specific code, for example, “**donor A‐S1**” describes donor A and Sample 1, whereas donor A‐S3 would be the code for the third stool donated by Donor A.

### 2.1 Anthocyanin Content and Composition of Black Rice and Bilberry Extracts

In the black rice extract, six anthocyanins were detected and, of these, four were identified (**Figure** **S3**, Supporting Information). The standard addition method (**Figure** **S4**, Supporting Information) was used to quantify the predominant black rice anthocyanin (Cy3Glc). The concentration of Cy3Glc was 333.6 ± 15.3 mg g^−1^ (33.3% w/w) dry weight black rice powder. In bilberry, however, 15 different anthocyanins were detected and identified, and 14 anthocyanins were quantified by using external standard curves of Cy3Glc, Dp3Glc, Mv3Glc, Pn3Glc, and Pt3Glc. The concentration of each bilberry anthocyanin, with total anthocyanin being calculated as the sum of the individual anthocyanin contents of 262.9 ± 5.46 mg g^−1^ (26.3% w/w) dry weight of bilberry powder (**Figure** **S5**, Supporting Information).

### 2.2 Degradation of Anthocyanins in the Human Colon Model

**Figure 2:**
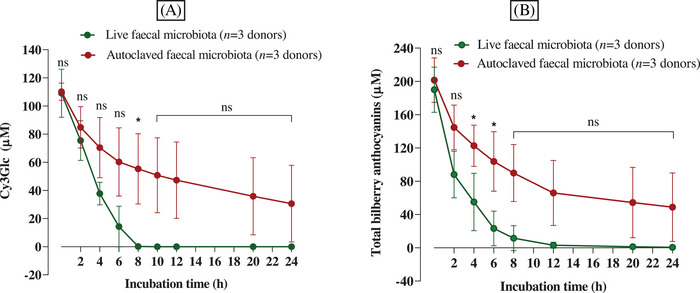
Degradation of black rice (A) and bilberry (B) anthocyanins over time was partly spontaneous and partly due to the gut microbiota. Black rice extract (18 mg powder, containing 6 mg of Cy3Glc) and bilberry extract (46 mg powder, containing 12.1 mg of total anthocyanins) were dissolved in 1 mL water, filtered, and immediately added to colon model vessels pre‐filled with sterile media (89 mL) and human fecal slurry (10 mL of a 10% slurry from a fresh stool) to give a final volume of 100 mL and a Cy3Glc concentration of 133.60 µM (60 µg mL^‐1^). Similar vessels were prepared but containing autoclaved fecal slurry rather than fresh fecal slurry. Control vessels contained fresh fecal inoculum and media, but no black rice or bilberry extracts. Incubations were carried out at pH 6.6–7.0 and 37 °C, over 24 h. Samples (0.5 mL) were collected at the times shown in the figure, mixed with 0.5 mL of 4% v/v aqueous formic acid, and after sample preparation, analyzed using HPLC‐DAD to determine the Cy3Glc concentration for black rice and total anthocyanin concentration for bilberry. The data shown are for three replicate incubations for each condition using single donor fecal samples: for black rice, donor A‐S1 (*n* = 1), donor B‐S1 (*n* = 1), and donor E‐S1 (*n* = 1), while for bilberry donor A‐S2 (*n* = 1), donor C‐S1 (*n* = 1), and donor F‐S1 (*n* = 1). No anthocyanins were detected in control vessels lacking black rice or bilberry extracts (data not shown). Values represent means ± SD. Statistical analysis was carried out with one‐way ANOVA with Tukey multiple comparisons for each time point and * *p* < 0.05.

In the vessels supplemented with black rice extract, the initial concentrations (*C*_initial_) of Cy3Glc in the 0 h samples were 109 ± 17.1 and 110.2 ± 6.1 µM in vessels containing fresh fecal slurry and autoclaved fecal slurry, respectively (**Figure** **2A**). Compared to the theoretical concentration (*C*_theo_) of inoculated Cy3Glc (133.4 µM), the *C*_initial_ was significantly lower with 18.4% and 17.5% reduction from sample with fresh fecal slurry and autoclaved fecal slurry, respectively. In bilberry‐treated vessels, 14 different anthocyanins were detected in colon model samples, corresponding to the 14 quantifiable anthocyanins determined in the extract. The *C*_initial_ of the total bilberry anthocyanins at 0 h in the presence of fresh and autoclaved fecal inocula were 190 ± 27.2 and 201.5 ± 26.7 µM, respectively, which were also lower than the *C*_theo_ (265 µM) (Figure 2B). An independent experiment (**Figure** **S6**, Supporting Information) showed that there was a rapid loss of anthocyanins (≈12%) within 1 min of adding anthocyanins to the fermentation vessel (1 min is the average time that passed between adding the anthocyanin extract to the colon model vessel and taking the first sample).

Another observation was that the fecal matrix only modestly affected the recovery of anthocyanins (**Figure** **S7**, Supporting Information). This is an important point because another process by which anthocyanins may ``disappear’’ is via adsorption to components in the fecal matrix such as fiber or bacteria. The data in Figure S7, Supporting Information are not consistent with this being an important process because i) there were minimal differences in the slopes between standard curves in the presence versus the absence of fecal matter, and ii) there was no evidence that later injected standards in a fecal matrix had lower than expected concentrations of anthocyanins. Although it was observed that the pellet formed after centrifuging samples taken from colon model vessels was a darker red color than the supernatant, which would be consistent with the reduction of anthocyanin concentration in the supernatant being due to some of the anthocyanin being bound to precipitated components in the pellets, this appears to only account for a tiny fraction of the total anthocyanins in a fermentation vessel. In addition, the fecal matrices might also negatively affect anthocyanin detection on DAD. This observation is in agreement with others^[^ ^17^, ^19^ ^]^ who showed lower recoveries of spiked anthocyanins when in fecal matrix due to irreversible attachment to fecal materials, such as proteins. Therefore, it was very important to match the same fecal matrices during the preparation of the external standard curves.

No anthocyanins were detected in any samples taken from the control vessels that had not been supplemented with black rice or bilberry extract. In the vessels containing black rice extract, Cy3Glc declined significantly faster in the presence of fresh fecal slurry, with an initial degradation rate (*k*_deg(0–2 h)_) of 16.8 ± 7.8 µM h^−1^, than in vessels containing autoclaved fecal slurry with *k*_deg(0–2 h)_ of 12.7 ± 5.2 µM h^−1^ (Figure 2A). At 24 h, Cy3Glc was still detectable at 30.8 ± 27.3 µM in vessels containing autoclaved fecal slurry, but it completely disappeared after 8 h in vessels containing fresh fecal slurry. It is worth noting that when we conducted fermentations that were similar except that anaerobic conditions were achieved using an anaerobic cabinet and there was no automated control of pH, we observed much slower loss of Cy3Glc over fermentation time, and this was associated with significant reductions in the fermentation media pH over time (**Figure** **S8**, Supporting Information). This is in keeping with anthocyanins being more stable at lower pH, but crucially both spontaneous and microbiota‐dependent processes are still involved. The concentration of Cy3Glc in the presence and absence of live microbiota was statistically significant at 8 h (*p* < 0.05). In addition, the concentration of bilberry anthocyanins declined faster in the presence of live fecal microbiota (*k*_deg(0–2 h)_ of 51 ± 13.8 µM h^−1^) compared to autoclaved fecal microbiota (*k*_deg(0–2 h)_ 28.4 ± 13.4 µM h^−1^), and this difference was statistically significant at 4 and 6 h (*p* < 0.05) (Figure 2B).

There was a substantial difference in the variance observed for spontaneous‐only versus spontaneous plus microbiota‐dependent ( = total) degradation of anthocyanins (Figure 2A). The average standard deviations for spontaneous only and spontaneous plus microbiota dependent degradation were 22.2 µM (0–24 h) versus 13.4 µM (0–6 h) (*p* < 0.05).

These data showed that the colonic degradation of Cy3Glc is partly microbiota‐dependent and partly independent of activity of the fecal microbiota. It is widely known that anthocyanins are not stable at near neutral pH^[^ ^21^ ^]^ and the non‐microbiota‐dependent component of Cy3Glc disappearance likely reflects chemical instability and will henceforth be referred to as “spontaneous degradation.” Spontaneous degradation was also observed during the incubation of black rice and bilberry anthocyanins in colon model experiments where no fecal slurry was added (**Figure** **S9**, Supporting Information).

### 2.3 Intra‐Individual Variations in Black Rice Anthocyanin Degradation Rates

**Figure 3:**
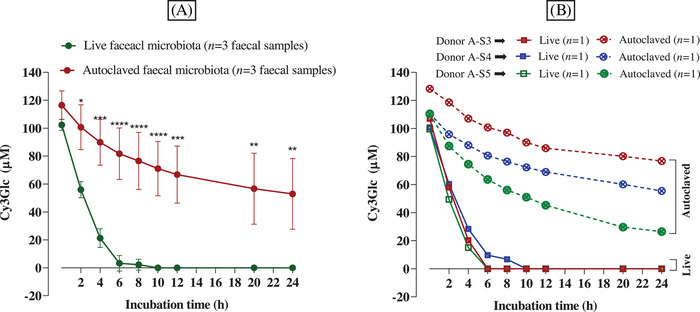
ntra‐individual variations in the loss of black rice anthocyanins (Cy3Glc) over 24 h. Black rice extract (18 mg powder, containing 6 mg Cy3Glc) was dissolved in 1 mL water, filtered, and immediately added to a colon model vessel pre‐filled with sterile media (89 mL) and human fecal slurry (10 mL of a 10% slurry from a fresh stool) to give a final volume of 100 mL and a Cy3Glc concentration of 133.60 µM (60 µg mL^‐1^). Similar vessels were prepared but containing autoclaved fecal slurry rather than fresh fecal slurry. Control vessels were prepared by incubating fresh fecal inoculum in the same colon model media but without black rice extract. Incubations were carried out at pH 6.6–7.0 and 37 °C, over 24 h. Samples (0.5 mL) were collected at the times shown in the figure, mixed with 0.5 mL of 4% v/v aqueous formic acid, and after sample preparation, analyzed using HPLC‐DAD to determine the Cy3Glc concentration. No Cy3Glc was detected in control vessels lacking black rice extract (data not shown). The experiments were carried out by using three different fecal samples from the same donor in separate experiments. In (A), data are presented as average from three different faecal samples (*n* = 3, values presented as means ± SD), while figure (B) presents the same data but showing each individual time course. Statistical analysis was carried out with one‐way ANOVA with Tukey multiple comparisons for each time point and **** *p* < 0.0001; *** *p* < 0.001, ** *p* < 0.01, * *p* < 0.05.

Three fecal samples were collected from the same donor on three different days, and each fecal sample was used to inoculate a colon model vessel (*n* = 1 vessel per fecal sample). One vessel per donor fecal sample was deemed adequate because we have shown that the technical variation (i.e., variation between three vessels set up with the same conditions and inoculated with the same donor fecal sample) was low; the average standard deviation across three replicate samples taken from each of three donors across 0–6 h was 4.7 µM (data presented in Section 3.4). At 0 h, the *C*_initial_ of Cy3Glc was 102.4 ± 3.9 and 116.4 ± 10.3 µM in the presence of live fecal microbiota and of autoclaved fecal microbiota, respectively (**Figure** **3**). The average *k*_deg(0–2 h)_ of Cy3Glc in the presence of live fecal microbiota was significantly faster (23.2 ± 2.4 µM h^−1^) than in the autoclaved fecal microbiota (7.9 ± 6.5 µM h^−1^), and it was statistically significant at 2 h (*p* < 0.05), 4 h (*p* < 0.001), 6–10 h (*p* < 0.0001), 12 h (*p* < 0.001), and 20–24 h (*p* < 0.01) (Figure 3A).

In addition, the *k*_deg(0–2 h)_ of Cy3Glc was similar when the models were inoculated with all three fresh fecal slurries from donor A (A‐S3, A‐S4, and A‐S5) (24.4, 20.1, and 25.1 µM, respectively), and no Cy3Glc was detected after 6 h in samples incubated with stool samples of donors A‐S3 and A‐S5 (Figure 3B). Only a very low concentration of Cy3Glc (6.7 µM) was detected in the 8 h sample which was collected from the vessel inoculated with the fecal sample from donor A‐S4.

Cy3Glc was detectable in all samples collected over the 24 h incubation from vessels that had autoclaved fecal slurries. Although the *C*_initial_ of Cy3Glc in samples inoculated with autoclaved stool slurries from A‐S4 and A‐S5 was almost the same (110.7 and 110.3 µM, respectively), the spontaneous *k*_deg(0–2 h)_ was slightly different, being 7.4 and 11.4 µM h^−1^, respectively (Figure 3B). At 24 h, the Cy3Glc concentration was significantly different when comparing both samples A‐S4 (55.5 µM) and A‐S5 (26.5 µM), showing that the fecal matrices affected the spontaneous degradation. In addition, the spontaneous degradation in the presence of autoclaved fecal slurries from fecal samples A‐S3 and A‐S4 was similar with *k*_deg(0–2 h)_ of 4.8 and 7.4 µM h^−1^, respectively.

There was a substantial difference in the variance observed for spontaneous‐only versus spontaneous plus microbiota‐dependent ( =total) degradation of anthocyanins between different fecal samples from the same donor (Figure 3). The average standard deviations for spontaneous only and spontaneous plus microbiota dependent degradation were 18.7 µM (0–24 h) versus 5.5 µM (0–6 h) (*p* < 0.0005).

### 2.4 Inter‐Individual Variation in Black Rice Anthocyanin Degradation Rates

**Figure 4:**
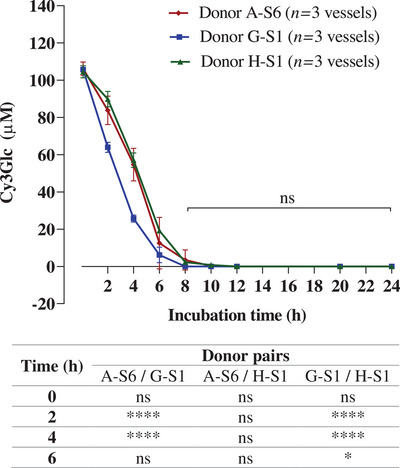
Inter‐individual variations in the loss of black rice anthocyanins (Cy3Glc 133.6 µM) over 24 h incubated with fresh fecal microbiota. Black rice extract (18 mg powder, containing 6 mg Cy3Glc) was dissolved in 1 mL water, filtered, and immediately added to a colon model vessel pre‐filled with sterile media (89 mL) and human fecal slurry (10 mL of a 10% slurry from a fresh stool) to give a final volume of 100 mL and a Cy3Glc concentration of 133.60 µM (60 µg mL^−1^). Control vessels were prepared by incubating fresh fecal inoculum in the same colon model media but without black rice extract. Incubation was carried out at pH 6.6–7.0 and 37 °C, over 24 h. Samples (0.5 mL) were collected at the times shown in the figure, mixed with 0.5 mL of 4% v/v aqueous formic acid, and after sample preparation, analyzed using HPLC‐DAD to determine the Cy3Glc concentration. No Cy3Glc was detected in control vessels lacking black rice extract (data not shown). The experiments were carried out using fecal samples from three different donors. Each fecal sample was incubated in triplicate. Values represent means ± SD. Statistical analysis was carried out with one‐way ANOVA with Tukey multiple comparisons for each time point and **** *p* < 0.0001; *** *p* < 0.001, ** *p* < 0.01, * *p* < 0.05.

Three different fecal samples were collected on the same day from three different donors (A‐S6, G‐S1, and H‐S1), and each fecal sample was used to inoculate three colon model vessels (*n* = 9 vessels). Cy3Glc was only detected in vessels containing black rice extract, and the *C*_initial_ means for fecal sample A‐S6, G‐S1, and H‐S1 were 106.2, 105.7, and 104.5 µM, respectively, which were lower than *C*_theo_ of 133.4 µM (**Figure** **4**).

In the presence of live fecal microbiota, the *k*_deg(0–2 h)_ of Cy3Glc differed between donors (11.2 ± 2.8, 20.9 ± 1.0 and 7.3 ± 1.8 µM h^−1^ for donors A‐S6, G‐S1, and H‐S1, respectively). Cy3Glc completely disappeared in the 8 h samples incubated with live fecal microbiota from G‐S1. In contrast, Cy3Glc was still detectable at 10 h in samples from both A‐S6 and H‐S1 although at very low concentrations. There were statistically significant differences in microbiota‐dependent degradation of Cy3Glc between donors A‐S6/G‐S1 at 2 and 4 h as well as between donors G‐S1/H‐S1 at 2, 4, and 6 h, whereas no statistically significant difference was shown between donors A‐S6/H‐S1.

There were also inter‐individual differences in the loss over time of total bilberry anthocyanins which is shown in Figure 2B. In contrast to what was observed with Cy3Glc from black rice extract, there was not a large difference in the variance between the with fresh versus with autoclaved fecal inoculum.

### 2.5 Effect of B‐Ring Substitution on Colonic Degradation of Bilberry Anthocyanins

**Figure 5:**
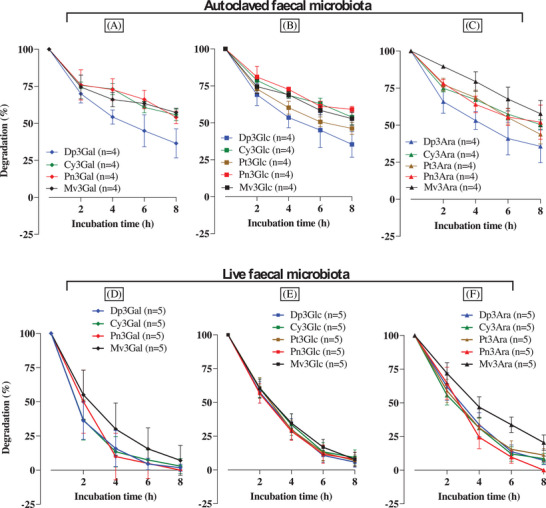
Effect of B‐ring on the loss of bilberry anthocyanins which are attached to the same sugar moiety (Galactose (Gal), glucose (Glc), or arabinose (Ara)) over the first 8 h in the autoclaved fecal microbiota (A, B, C) and in the presence of live faecal microbiota (D, E, F). Bilberry extract (46 mg, containing 12.1 mg anthocyanins) was dissolved in 1 mL water, filtered, and immediately added to a colon vessel pre‐filled with sterile media (89 mL) and human fecal slurry (10 mL of a 10% slurry from a fresh stool) to give a final volume of 100 mL and a total anthocyanin concentration of 265 µM (120 µg mL^−1^). Incubations were carried out at pH 6.6–7.0 and 37 °C with continuous nitrogen flow. Samples (0.5 mL) were collected at the times shown in the figure, mixed with 0.5 mL of 4% v/v aqueous formic acid, and after sample preparation, analyzed using HPLC‐DAD to determine the concentrations of 14 individual anthocyanins. All concentrations were normalized to 100% of the initial quantified concentration at 0 h. The data shown are for five replicates of fresh faecal samples and four replicates of autoclaved faecal samples using donor fecal samples from donor A (A‐S2), donor C (C‐S1 and C‐S2), and donor F (F‐S1 and F‐S2). Values represent means ± SD. A) and D) Loss of bilberry anthocyanins conjugated with galactose. B) and E) Loss of bilberry anthocyanins conjugated with glucose. C) and F) Loss of bilberry anthocyanins conjugated with arabinose.

Bilberry extract contained 14 different anthocyanins including multiple anthocyanidins and different glycosylation patterns, therefore, it was possible to assess if these structural differences affected the *k*_deg_ of anthocyanins. In the absence of live microbiota, all bilberry anthocyanins spontaneously degraded over time (**Figure** **5**A–C). However, the rate of loss was considerably faster in the presence of live microbiota (Figure 5D–F).

The substation pattern of the B‐ring of the anthocyanidin moiety appeared to play a role in anthocyanin degradation, especially on spontaneous degradation. The differences in the rates of spontaneous degradation between different bilberry anthocyanins were quite substantial, for example, Dp‐based anthocyanins (Dp3Gal, Dp3Glc, and Dp3Ara) degraded substantially faster than the other anthocyanins. Mv3Ara was degraded the slowest of all the investigated anthocyanins. In the presence of live microbiota there were only modest differences in the relative rates of loss of different anthocyanins (Figure 5D–F), and these were not statistically significant. For example, five different anthocyanidins (Dp, Cy, Pt, Pn, and Mv) conjugated with Glc had very similar *k*_deg(0–2 h)_ and overall degradation patterns over 8 h incubation (Figure 5E). Similarly, Dp, Cy, Pt, and Pn, conjugated with Ara had very similar *k*_deg(0–2 h)_, although Mv was not only more stable in the first 2 h, but also over 8 h incubation.

### 2.6 Effect of Different Sugars in the 3‐*O*‐Glycosylation of Bilberry Anthocyanins on Their Colonic Degradation

**Figure 6:**
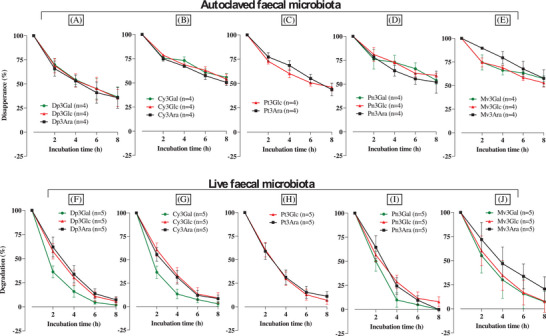
Effects of different sugar substitutions (galactose [Gal], glucose [Glc], or arabinose [Ara]) on the loss of individual bilberry anthocyanins over the first 8 h in the presence of autoclaved fecal microbiota (A–E) and in the presence of live fecal microbiota (F–J). Bilberry extract (46 mg, containing 12.1 mg anthocyanins) was dissolved in 1 mL water, filtered, and immediately added to a colon vessel pre‐filled with sterile media (89 mL) and human fecal slurry (10 mL of a 10% slurry from a fresh stool) to give a final volume of 100 mL and a total anthocyanin concentration of 265 µM (120 µg mL^−1^). Incubations were carried out at pH 6.6–7.0 and 37 °C with continuous nitrogen flow. Samples (0.5 mL) were collected at the times shown in the figure, mixed with 0.5 mL of 4% v/v aqueous formic acid, and after sample preparation, analyzed using HPLC‐DAD to determine the 14 individual anthocyanin concentrations. All concentrations were normalized to 100% of the initial quantified concentration at 0 h. The data shown are five replicates using donor fecal samples from donor A (A‐S2), donor C (C‐S1 and C‐S2), and donor F (F‐S1 and F‐S2). Values represent means ± SD. A and F) Loss of the delphinidin glycosides from the bilberry extract. B and G) Loss of cyanidin glycosides from the bilberry extract. C and H) Loss of the petunidin glycosides from the bilberry extract. D and I) Loss of peonidin glycosides of bilberry anthocyanins. E and J) Loss of malvidin glycosides from the bilberry extract.

Different sugar moieties of anthocyanins showed a very modest effect on the spontaneous degradation of bilberry anthocyanins (**Figure** **6**A–E), whereas a considerable effect was shown of the microbiota‐dependent degradation (Figure 6F–J). Anthocyanins containing a galactose moiety exhibited faster microbiota‐dependent degradation (for Dp, Cy, Pn, and Mv) than those substituted with other sugars, whereas those containing an arabinose moiety were degraded relatively slowly. This may be because there is a difference in the ability of fecal microbiota to hydrolyze arabinose substitutions compared to galactose and glucose.

## 3 Discussion

Evidence from epidemiological, human dietary intervention, and pre‐clinical studies suggests that anthocyanin consumption is associated with a reduction in disease risk and causes beneficial changes in biomarkers for various cardiometabolic and other diseases.^[^ ^6^, ^7^, ^8^, ^12^ ^]^ However, the bioavailability of anthocyanins is very low and it has been postulated that the majority of ingested anthocyanins are metabolized via bacterial fermentation and subsequently absorbed from the colon,^[^ ^15^, ^16^ ^]^ and it is these degradation products that interact with host cells and tissues to deliver the benefits. The notion that the major circulating metabolites of anthocyanins are ring‐fission products was confirmed in the report of De Ferrars et al.,^[^ ^14^ ^]^ but the origin of these ring‐fission products remains unknown. The study of colonic metabolism of anthocyanins presented here has shown that i) the colonic degradation of anthocyanins was partly spontaneous and partly due to the activity of the human gut microbiota, ii) microbiota‐dependent degradation was subject to high inter‐individual variations and modest intra‐individual variations, iii) spontaneous degradation showed even higher intra‐individual variations, and iv) B‐ring substitution patterns and glycosylation affected the rates of both spontaneous and gut microbiota‐dependent degradation.

**Figure 7:**
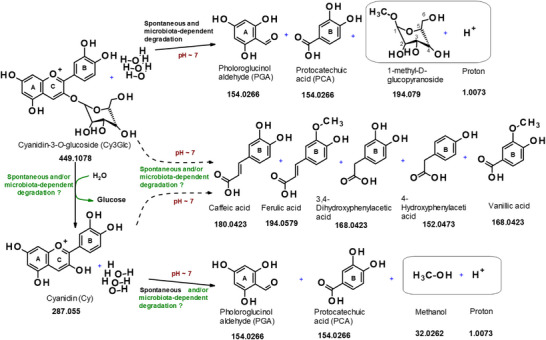
Schematic representation of the currently known degradation steps and metabolites of Cy3Glc metabolism. All the metabolites shown here are known to be derived from Cy3Glc after oral ingestion by humans, except those inside boxes which we tentatively propose here to be generated but there is not currently direct evidence to support this. The proposal that 1‐methyl‐D‐glucopyranoside and a proton are co‐products that would be generated along with PCA and PGA from Cy3Glc, and that methanol and a proton are co‐products that would be generated along with PCA and PGA from Cy are based on balancing each of the chemical equations. Metabolic steps that are known to occur via spontaneous and/or microbiota‐dependent processes are accompanied by bold black text. The solid arrows represent the data extracted from the in vitro reports (Hanske et al.^[^ ^17^, ^18^, ^19^, ^20^, ^25^ ^]^; Kay et al.^[^ ^21^ ^]^; and Woodward et al.^[^ ^21^, ^22^ ^]^), while the dotted arrows represent data extracted from the in vivo report (De Ferrars et al.^[^ ^14^ ^]^). Steps where it is not known if they are due to spontaneous or gut microbiota‐dependent processes are accompanied by bold green text. Numbers below each compound are the accurate mass to four decimal places.

We have shown for the first time that colonic degradation of anthocyanins is a consequence of both spontaneous processes (not requiring live gut microbes) and gut microbiota‐dependent processes. Prior to this report, the literature contained accounts of two types of studies of the degradation of anthocyanins/anthocyanidins in relation to human metabolism and bioavailability. First, there are reports of the spontaneous degradation of various anthocyanins/anthocyanidins in aerobic, near‐neutral pH conditions, and the appearance of small phenolic compounds apparently derived from fission of the C‐ring. The most commonly reported ring‐fission products for Cy3Glc/Cy are the B‐ring phenolic acid protocatechuic acid (PCA) and the A‐ring phenolic aldehyde phloroglucinol aldehyde (PGA).^[^ ^21^, ^22^ ^]^ The second study was by Hanske et al.^[^ ^20^ ^]^ who reported the appearance of PCA and PGA when Cy3Glc was fermented under anaerobic conditions in the presence of human feces. Based on this limited data, it would be easy to conclude that both the spontaneous and gut microbiota‐dependent processes generate the B‐ring PCA (C6‐C1) and the A‐ring PGA (C6‐C1) from the same ring‐fission reaction (**Figure** **7**). However, the list of [^13^C]‐labeled metabolites that were shown to appear in human plasma and urine following ingestion of 500 mg of penta‐[^13^C] Cy3Glc included compounds with C6‐C3 (ferulic acid, caffeic acid) and C6‐C2 (3,4‐dihydroxyphenylacetic acid and 4‐hydroxyphenylacetic acid) structures^[^ ^14^ ^]^ showing that other ring fission processes also appear to be important. There are a limited number of studies of anthocyanin degradation in the human colon (e.g., Gonzalez‐Barrio et al.^[^ ^23^ ^]^ and Bresciani et al.^[^ ^24^ ^]^) and there remain important gaps that should be addressed by future research including i) determining the step‐by‐step chemical processes that occur during both spontaneous and microbiota‐dependent breakdown of Cy3Glc and other anthocyanins, ii) identifying the intermediates and the end products of these two processes, and iii) attempting to connect these to the established human metabolites^[^ ^14^ ^]^ by considering, for example, human phase‐2 metabolism when analyzing samples from human intervention studies.

Results presented in this report show a significant role of spontaneous chemical processes in the degradation of anthocyanins in the human colon. Various factors affect anthocyanin stability, in particular the pH of the medium/intestinal milieu, because anthocyanins are less stable at near‐neutral pH.^[^ ^21^ ^]^ We have shown that the spontaneous degradation of anthocyanins occurred in conditions mimicking the human colon (anaerobic, neutral, pH). Prior to this study, reports of the spontaneous degradation of anthocyanins were based on aerobic conditions^[^ ^17^, ^18^, ^19^, ^20^, ^25^ ^]^ or the possible contribution of spontaneous degradation was not investigated.^[^ ^26^ ^]^ For example, some investigated spontaneous degradation by simply incubating anthocyan(id)ins aerobically in phosphate buffered solutions,^[^ ^21^, ^22^ ^]^ which is not representative of the anaerobic conditions of the colon. Furthermore, there were also discrepancies between reports that described aerobic spontaneous degradation of anthocyanins/anthocyanidins. For example, Kay et al.^[^ ^21^ ^]^ reported that the recovery of PCA and PGA was equivalent to loss of Cy (on the basis that stoichiometric conversion of one mole of Cy3Glc which gave rise to one mole each of the PCA and PGA), whereas Woodward et al.^[^ ^22^ ^]^ reported 100% (within 2 h) and 85% (within 24 h) loss of Cy and Cy3Glc but PCA only accounted for 39 and 12 mol% of the initial concentration of Cy and Cy3Glc, respectively, suggesting that other breakdown products were also being generated. It is possible that the degraded Cy3Glc/Cy that was not converted to PCA and PGA could have formed other degradation products, for example, the C6‐C3 and C6‐C2 type metabolites identified in human plasma and urine by De Ferrars et al.^[^ ^14^ ^]^ Another interesting feature of the aerobic spontaneous degradation of Cy data reported by Woodward et al.,^[^ ^22^ ^]^ was that PCA continued to accumulate up to 24 h even though Cy had completely disappeared within 2 h, providing strong evidence of intermediate(s).

Indeed, a number of important questions remain with regard to the spontaneous degradation of anthocyan(id)ins under both aerobic and anaerobic conditions. For example, what is the fate of d‐glucose? It seems unlikely that a de‐glycosylation process could occur at neutral pH in the absence of a glucosidase, and if so, in order to balance the chemical equation one of the products would likely be 1‐methyl‐O‐beta‐d‐glucose and a proton (Figure 7). Further, during spontaneous degradation of Cy3Glc it is not known if the C‐ring opening is the first step, but this would generate a colorless intermediate and be consistent with the loss of Cy3Glc occurring much more rapidly than the appearance of A‐ and C‐ring‐derived fission products. In addition, it is not known how the C6‐C3 (hydroxycinnamic acid) and C6‐C2 (phenylacetic acid) type breakdown products reported to be generated from penta‐[^13^C]‐Cy3Glc^[^ ^14^ ^]^ are formed, and whether this is a spontaneous or gut microbiota‐dependent process (Figure 7).

We have shown that spontaneous degradation of anthocyanins occurs in anaerobic conditions throughout the 24 h fermentations and is a substantial contributor to the total disappearance of anthocyanins (Figures 2 **and** 3, **Table** **1**). An interesting observation was the substantial variation in the rates of anthocyanin degradation observed between colon model incubations carried out with autoclaved stools collected from the same donor but on different days (Figure 3) exhibited similar variance to that observed for stool samples collected from different donors (Figure 2). The significant variation we observed for the single donor might be driven by substantial differences in the microbiota composition between collected stools or it might be a consequence of the spontaneous degradation of anthocyanins being very sensitive to relatively small changes in the fecal matrix. It is likely that several of variables could have contributed to the observed variation, including what had been consumed by the donor over the preceding 1–2 days and the consistency of the stool itself. Given that the inter‐ and intra‐individual variance in the rates of degradation of anthocyanins incubated with autoclaved fecal inoculates were similar, it is possible that the fecal matrix rather than the fecal microbiome is the most important factor explaining between‐stool variance.

**Table 1:** Estimated rates of spontaneous and gut microbiota‐dependent degradation of black rice anthocyanin Cy3Glc

Black rice Cy3Glc					
	Concentration [µM]		Δ concentration [µM]		
Incubation time [h]	Autoclaved stools	Fresh stools	Spontaneous degradation	Microbial degradation	Total degradation
0 (C_initial_)	110.2	109.0	00.0 (00.0%)	00.0 (00.0%)	00.0 (00.0%)
2	84.8	75.4	25.4 (75.6%)	08.2 (24.4%)	33.6 (30.8%)
4	70.4	37.7	39.8 (55.8%)	31.5 (44.2%)	71.3 (65.4%)
6	60.2	14.3	50.0 (52.8%)	44.7 (47.2%)	94.7 (86.9%)
8	55.3	00.0	54.9 (50.4%)	54.1 (49.6%)	109.0 (100%)

The spontaneous degradation value (e.g., at 2 h) is the concentration difference in the presence of autoclaved microbiota at 0 and 2 h. Whereas, the microbiota‐dependent degradation value is the concentration difference in the presence of live microbiota at 0 and 2 h, and subtracting the spontaneous degradation at 2 h (e.g., (109‐75.4)‐25.4)). The values in parentheses denote the change in concentration of Cy3Glc as a percentage (%) of the concentration in the first (0 h) timepoint sample.

There are some existing reports that suggest spontaneous degradation of anthocyanins is limited or does not occur at all. These studies involved the use of simple buffer solutions^[^ ^25^, ^27^ ^]^ or have utilized filtered human fecal samples,^[^ ^18^, ^19^ ^]^ neither of which closely mimic the complex matrix in the colon. Indeed, it would be possible that multiple factors would significantly affect colonic spontaneous degradation of anthocyanins as we show in the case of pH (**Figure** **S8**, Supporting Information), and the fermentation matrix (Figure 3B). In addition, there are considerable variations in the rates of microbiota‐dependent degradation of anthocyanins reported in the literature, and it is likely this is caused by differences in the types of media and pH that were used,^[^ ^18^, ^20^, ^28^ ^]^ in the different experimental models (e.g., in vitro colon models inoculated with human feces vs pig large intestinal contents,^[^ ^29^, ^30^ ^]^ feces from rats,^[^ ^26^ ^]^ or isolated bacterial strains^[^ ^27^, ^31^ ^]^), and the purity of the anthocyanins used.^[^ ^25^, ^26^, ^31^, ^32^ ^]^

The investigation of bilberry anthocyanin degradation goes beyond the existing reports in the literature where only single or few anthocyanin candidates were investigated in vitro.^[^ ^17^, ^18^, ^20^, ^30^ ^]^ We demonstrate that the B‐ring and different sugars in the 3‐*O*‐glycosylation affect the degradation rates of anthocyanins. We have shown that there are considerable differences in anthocyanin *k*_deg_ between different anthocyanin aglycones (i.e., Cy, Dp, Pn, and Mv) in both spontaneous and gut microbiota‐dependent degradation. For example, Mv showed more stability than the other anthocyanins whereas Dp showed less stability in both microbiota‐dependent and spontaneous degradation. As Avila et al., suggested, the larger the number of the hydroxyl group on the B‐ring, the lower the chemical stability of the anthocyanin.^[^ ^27^ ^]^ Consistent with this, we demonstrate that anthocyanins with more hydroxyl groups on the B‐ring are more susceptible to spontaneous degradation. In contrast, the presence of methoxy group on the B‐ring increases the chemical stability of anthocyanins. This suggests that the structure of B‐ring considerably affects the chemical stability of anthocyanins, and consequently is an important factor in the overall colonic degradation of anthocyanins.

The glycosylation of anthocyanins was shown to play an important role in the *k*_deg_ of anthocyanins, in particular the microbiota‐dependent degradation. For example, galactose (Gal) decreased the stability of anthocyanins, whereas arabinose (Ara) was shown to increase the stability of anthocyanins (Gal < Glc < Ara). Glucose is the only unmodified monosaccharide that exists in substantial quantities in living organisms, in which it exists primarily extracellularly.^[^ ^33^ ^]^ In addition, it was reported that Glc is more readily taken up by mammalian cells than Gal.^[^ ^34^ ^]^ Ryan et al.,^[^ ^35^ ^]^ reported that Glc was utilized more rapidly than Gal by cultures of 27 isolated oral bacteria. This suggested that it is easier for bacteria, including those of the gut microbiota, to consume Glc as a carbon source than other sugar moieties such as Gal. However, data show that the degradation of anthocyanidin galactosides was faster compared to the glycosylation with other sugars (e.g., glucosides). It is possible that gut microbes can hydrolyze anthocyanidin galactosides more readily than glucosides.

We also used a non‐pH‐controlled colon vessel to investigate the degradation of black rice Cy3Glc. Although the microbial *k*_deg(0–2)_ of black rice Cy3Glc was relatively similar between a pH‐controlled and a non‐pH‐controlled model, the overall degradation was significantly different; Cy3Glc was fully degraded within 6–8 h in pH‐controlled vessels (Figures 2, 3, 4), whereas it was not fully degraded in non‐pH‐controlled vessels after 24 h incubation (**Figure** **S8**, Supporting Information). It was observed that the pH dropped substantially in the presence of live microbes to ≈5.5 and 4.5 within 2 and 6 h, respectively, and the degradation was very slow after 6 h. This might be consistent with the production of significant quantities of organic acids, for example, short chain fatty acids (SCFAs), by the microbiota during the fermentation, which caused the reduction of the pH of the medium. In addition, no significant differences in the rates of anthocyanin disappearance were observed between experiments carried out with inactivated fecal inocula (autoclaved) in pH‐controlled colon models (``batch’’) and pH‐uncontrolled models (stirred bottles in anaerobic cabinet). This shows that when microbiota‐dependent organic acid production is prevented, the pH does not change over time and as a consequence there is no effect on anthocyanin spontaneous degradation.

A limitation of the work presented here is that in vitro colon models only reflect the conditions of the human colon, and the observations obtained from these experiments cannot reflect transformations that potentially occur in the upper gut, particularly the ileum, where the structure of the microbiota community differs,^[^ ^36^, ^37^ ^]^ and nor do they take account of digestive processes in the small intestine or absorptive processes that may draw compounds out of the gut lumen. With regard digestive processes, it is worth noting that neither gastric or pancreatic secretions contain β‐glucosidases/β‐galactosidases that could deglycosylate Cy3Glc or bilberry anthocyanidin glucosides/galactosides, and neither do they contain other glycosyl hydrolases that could remove other sugars from bilberry anthocyanins. Some spontaneous degradation of anthocyanidins may occur in the small intestine, but as discussed above, the data from Ferrars et al.^[^ ^14^ ^]^ clearly shows that the colon is the main site of production of ring fission products of anthocyanins, meaning the data presented here are relevant in vivo.

Future research should focus on delineating the step‐by‐step transformations occurring during both spontaneous and microbiota‐dependent anaerobic degradation of anthocyan(id)ins so that the degradation products can be identified, and metabolic routes determined. It will also be important to determine how molecular oxygen affects the degradation of anthocyanins, and specifically how it affects what breakdown products are generated compared to in the absence of oxygen. It would also be interesting to identify the major matrix factors that affect anthocyanin(id)in breakdown, considering the significant differences we observed between different stools from the same donor.

## 4 Conclusions

Here we demonstrate that i) the degradation of anthocyanins was partly spontaneous and partly due to the gut microbiota and ii) microbiota‐dependent degradation was subject to high inter‐individual variation and modest intra‐individual variation. These data show that the human gut microbiota is important for anthocyanin metabolism and supports the notion that anthocyanin microbiota metabolites are likely to contribute to the reported beneficial effects of consuming anthocyanins on human health.

## 5 Experimental Section

### Materials

All water used in this study was 18 MΩ cm^−1^ Milli‐Q water, and solvents were of high‐performance liquid chromatography (HPLC) grade. The anthocyanin‐rich extract powders prepared from black rice and bilberry were purchased from the Beijing Gingko Group (BGG), China. Kuromanin chloride (Cy3Glc), myrtillin chloride (delphinidin‐3‐*O*‐glucoside, Dp3Glc), peonidin‐3‐*O*‐glucoside chloride (Pn3Glc), oenin chloride (malvidin‐3‐*O*‐glucoside, Mv3Glc), and petunidin‐3‐*O*‐glucoside chloride (petunidin‐3‐*O*‐glucoside, Pt3Glc) were purchased from Extrasynthese (Genay, France). Other chemicals and reagents were purchased from Sigma (Sigma, Gillingham, UK) unless otherwise stated within the text.

### Determination of Anthocyanin Composition in Black Rice and Bilberry Extract Powders Using HPLC‐DAD‐MS

The quantification of anthocyanins was carried out using a series of concentrations of both black rice and bilberry extract powders (1, 0.5, 0.25, 0.125, 0.062 mg mL^−1^), which were prepared in acidified water with 2% formic acid (FA). Anthocyanin separation was performed via reverse phase HPLC using an Agilent 1100 series HPLC. 20 µL of each of the extract solutions were injected into the Kinetex XB‐C18 column (100 × 4.6 mm; particle size 2.6 µm) with a flow rate of 1 mL min^−1^ at 40 °C using 5% FA in water (eluent A) and 5% FA in acetonitrile (eluent B). The gradient was 5% mobile phase B with injection for 2 min, increased to 7% at 10 min, 10% at 15 min, 13% at 16 min, 20% at 18 min, and then re‐equilibrated to initial conditions over 6 min. Anthocyanin detection of individual anthocyanins was achieved using the DAD at a wavelength of 520 nm, and the quantification of bilberry anthocyanins was achieved by using external standard curves of five purified anthocyanin standards (Cy3Glc, Dp3Glc, Pn3Glc, Mv3Glc, and Pt3Glc) and quantifying using the standard curve of the equivalent anthocyanin, or where these were not available, the most similar available anthocyanin (e.g., all Dp‐glycosides against Dp3Glc). The standard addition method was used to quantify the predominant anthocyanin (Cy3Glc) in the black rice extract powder.

The identification of anthocyanins was based on i) having the same retention time (RT) as that of one of the five highly purified anthocyanin standards that were run alongside samples, ii) the presence of a signal corresponding to the mass of the parent anthocyanins, iii) the presence of known/predicted daughter ions of the anthocyanidins (e.g., masses of 287, 301, 331, 303, 317 corresponding to Cy, Pn, Mv, Dp, and Pt aglycones, respectively), and iv) the existing literature where the majority of the fragments identified were consistent with published data.^[^ ^38^ ^]^

### In Vitro Colonic Fermentations

The characteristics of the study participants were as described by Day‐Walsh et al.^[^ ^39^ ^]^ Briefly, stool samples were provided by volunteers who were from both genders, aged between 25 and 54, declared to be in a good health, had no diagnosed chronic gastrointestinal health problems, and had not consumed antibiotics for at least 6 months prior to the stool donation. Donors were not instructed to follow any particular diets or consume any particular foods and in keeping with the terms of the ethical approval, no metadata was collected. The stools were processed on the same day they were received. The study was approved by the local Quadram Institute Bioscience Human Research Governance committee (IFR01/2015), and by the London‐Westminster Research Ethics Committee (15/LO/2169) and the trial was registered at http://www.clinicaltrials.gov (NCT02653001). All participants gave their signed informed consent prior to donating samples. The study was carried out in accordance with the Declaration of Helsinki.

The fermentation experiments were carried out using a pH‐controlled in vitro human colon model as previously described by Parmanand et al.^[^ ^40^ ^]^ Briefly, 300 mL glass fermentation vessels were prepared and autoclaved before each experiment. The night before the experiment, 89 mL of autoclaved colon model media, containing constituents as reported by Day‐Walsh et al.,^[^ ^39^ ^]^ was transferred into colon model vessels equipped with a magnetic stirrer and continuously degassed with O_2_‐free nitrogen for equilibration of anaerobic conditions. On the day of the experiment, water baths that pumped heated water into jackets surrounding each vessel were switched on to establish a vessel temperature of 37 °C, and Fermac 260 pH control units were used to maintain a pH between 6.6 and 7.0 by adding either 0.5 M HCl or 0.5 M NaOH (**Figure** **S1**, Supporting Information).

### Incubation of Black Rice and Bilberry Anthocyanin Extracts in the Batch Colon Model

For inoculum preparation, a fresh fecal slurry with 10% fecal sample was prepared immediately after collection by diluting the fecal sample 10‐fold (1/10 w/v) with sterile phosphate buffered saline (PBS) (0.01 M), and then was homogenized in a stomacher (Seward, UK) for 45 s at 230 rpm. To study the chemical stability of anthocyanins, an autoclaved fecal slurry was prepared by autoclaving a portion of the fresh fecal slurry at 121 °C for 60 min to inactivate all viable microorganisms and denature enzymes.

For studying the microbial metabolism of anthocyanins, the colon model vessel was inoculated with 10 mL of the fresh fecal slurry, and then 18 mg of black rice extract powder (33.3% w/w Cy3Glc = 6 mg Cy3Glc) was dissolved in 1 mL water, filtered by a syringe filter (0.20 µm) and added to the vessels to give a final concentration of 133.3 µM (60 µg mL^−1^) of Cy3Glc in a total volume of 100 mL of 1% fecal inoculated colon media (**Figure** **S2**, Supporting Information). This amount was broadly consistent with the estimated daily intake of anthocyanins reported in epidemiological studies (≈20 mg day^−1^) reaching a colon with a volume of 0.5 L. For bilberry treatment, the same procedure was followed but 46 mg of bilberry extract powder (26.2% w/w of mixture of 14 anthocyanins = 12 mg total anthocyanins) was added in the place of the black rice powder. The control vessel was prepared by only adding 10 mL of the fresh fecal slurry and this was also used as the matrix for preparing standards/standard curves for HPLC‐DAD analysis. For studying the spontaneous degradation of anthocyanins, the colon model vessel was inoculated with 10 mL of autoclaved fecal slurry. Samples (500 µL) were collected at 0, 1, 2, 4, 6, 8, 12, 20, and 24 h and immediately added to 500 µL of acidified water (4% v/v FA) and stored at −20 °C prior to analysis.

### Quantification of Anthocyanins in Colon Model Samples

Colon model samples were thawed at room temperature for 1 h, vortexed, and then centrifuged at 17,000 × *g* for 10 min. 250 µL of supernatant was transferred into HPLC vials (with a 300 µL amber glass insert) and the anthocyanin concentrations were determined using the HPLC‐DAD method described in Section 3.2.

### Statistical Analysis

Statistical analysis was performed in GraphPad Prism (version 9.3 for Windows, GraphPad Software, La Jolla, CA, USA, www.graphpad.com). Statistical analysis was carried out using one‐way ANOVA with Tukey multiple comparisons for each time point and **** *p* < 0.0001; *** *p* < 0.001, ** *p* < 0.01, * *p* < 0.05.

## Conflict of Interest

The authors declare no conflict of interest.

## Author Contributions

E.S. contributed to experimental design, carried out the experimental work, data analysis & interpretation, and wrote the manuscript; P.D. contributed to experimental design and experimental work; L.K. and A.N. reviewed the manuscript; and P.K. provided original ideas and contributed to experimental design, data interpretation, and the writing of the manuscript. All authors read and approved the final manuscript.

## Supporting information

Supporting Information

## Data Availability

The data that support the findings of this study are available from the corresponding author upon reasonable request.
