# Supplementary material for: Spontaneous and Microbiota‐Driven Degradation of Anthocyanins in an In Vitro Human Colon Model
Source: Mol Nutr Food Res. 2023 Jul 31;67(19):2300036. doi: 10.1002/mnfr.202300036 (PMC10909555; doi:10.1002/mnfr.202300036)
Supplement: Supplementary file 1 — Supporting Information [file MNFR-67-2300036-s001.pdf]

Supplementary materials

Spontaneous and microbiota-driven degradation of anthocyanins in an in vitro human colon model

Emad Shehata[1, 2], Priscilla Day-Walsh[1,3,4], Lee Kellingray[1], Arjan Narbad[1], Paul A Kroon[1]*

[1]Quadram Institute Bioscience, Norwich Research Park, Norwich, NR4 7UQ, UK.

[2]Chemistry of Flavour and Aroma Dept, National Research Centre, 33 El Buhouth St., Dokki, 12622 Cairo, Egypt.

[3]Department of Obstetrics and Gynaecology, University of Cambridge, The Rosie Hospital, Robinson Way, Cambridge CB2 0SW, UK

[4]Centre for Trophoblast Research (CTR), Department of Physiology, Development and Neuroscience, University of Cambridge, Cambridge, CB2 3EG, UK.

*Corresponding author: paul.kroon@quadram.ac.uk

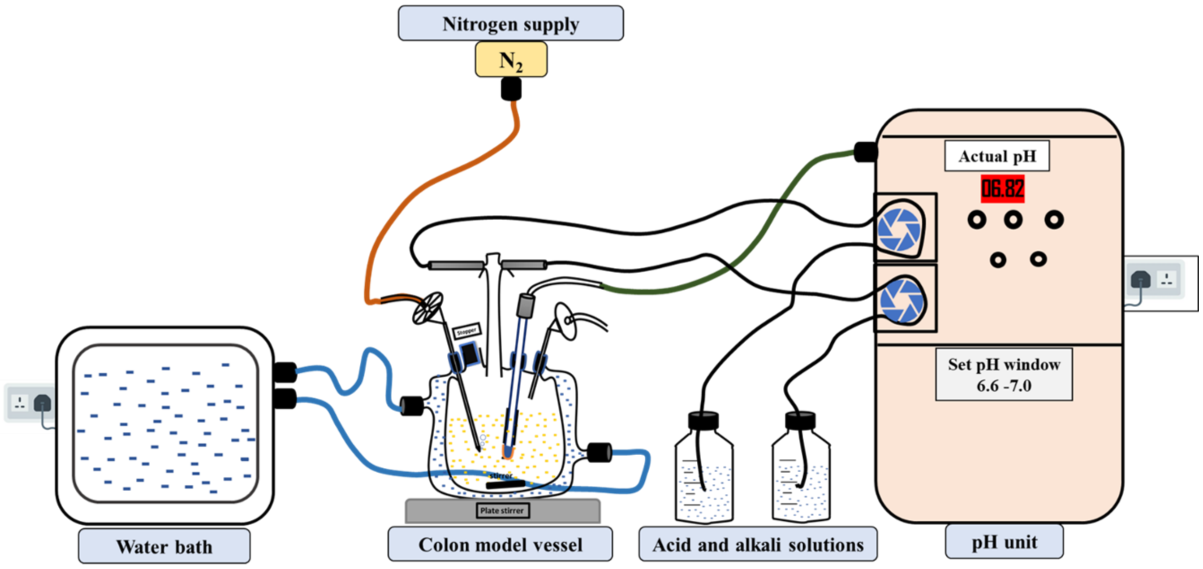

Supplementary Figure 1. Schematic of the human *in-vitro* colon model. The model was set up with the colon model vessel, the pH unit, acid & alkali solutions, nitrogen supply, and water bath.

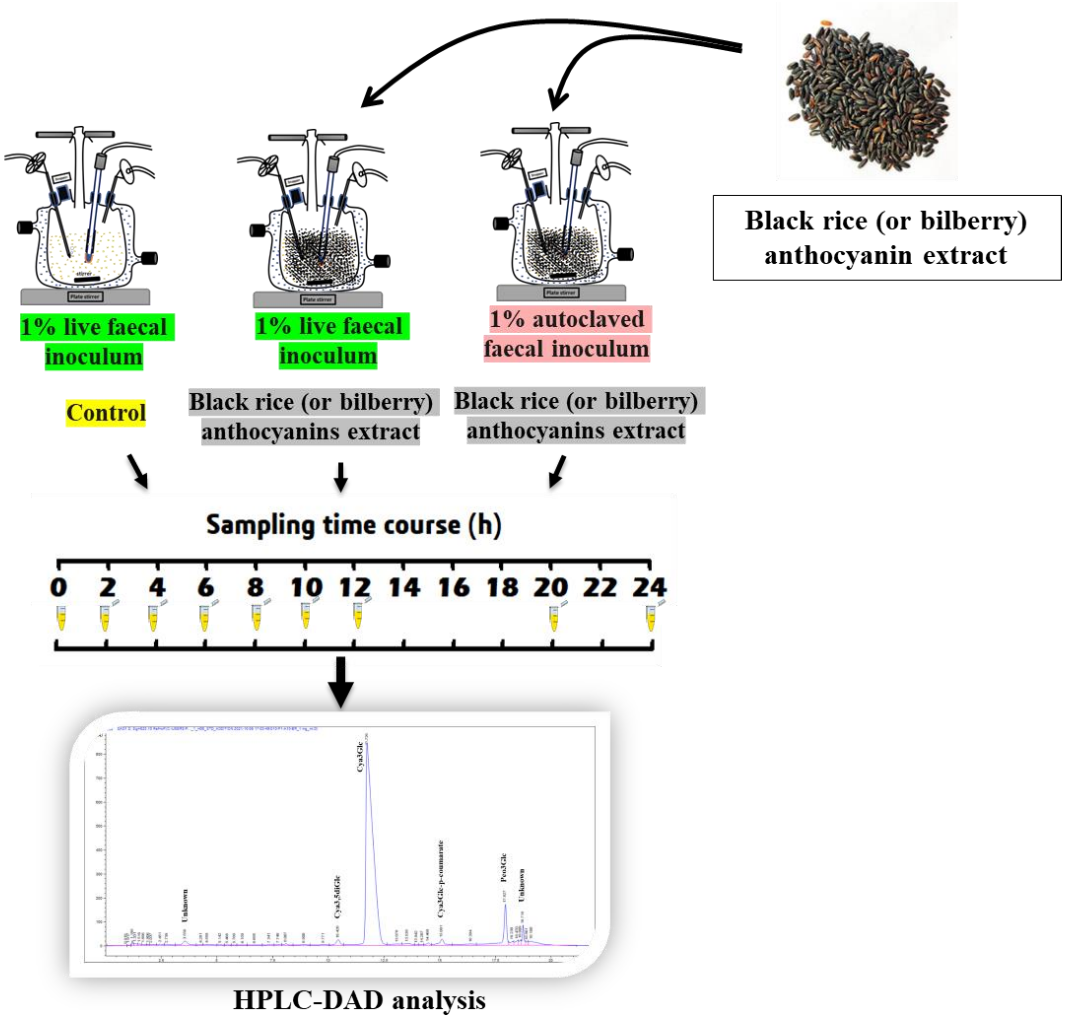

Supplementary Figure 2. Schematic of experimental design for investigating the metabolism of anthocyanins by the human faecal gut microbiota.

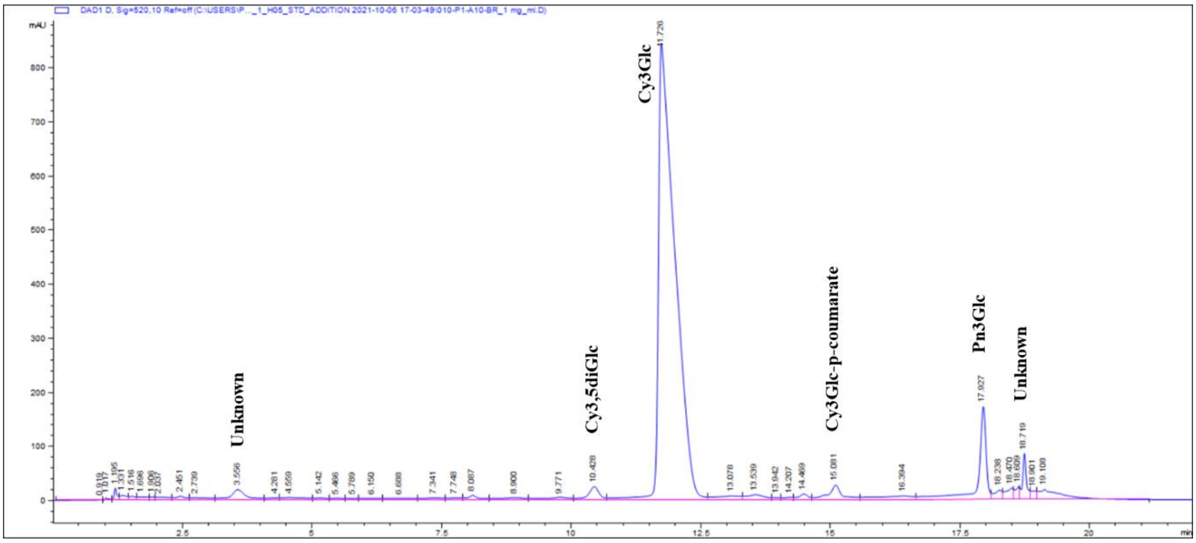

Peak	RT DAD$_{520nm}$ (min)	**Parent ion** TOF-MS [M+H]$^{+}$	**Other ions** TOF-MS [M+H]$^{+}$	**Identified anthocyanin**	**Content**[#] (mg g^{-1} d.w.)	**% of total anthocyanins**
**1**	3.55	737.170	575.118, 423.073, 287.058	Unknown (Cy-type anthocyanin)	4.68 ± 0.08 [a]	1.22
**2**	10.42	611.160	449.108, 287.058	Cya3,5diGlc	5.98 ± 0.07 [a]	1.56
**3**	11.72	449.110	287.058	Cy3Glc	333.44 ± 5.4	87.21
**4**	15.08	595.166	287.057	Cy3Glc-*p*-coumarate	6.26 ± 0.18 [a]	1.64
**5**	17.92	463.124	301.073	Pn3Glc	23.48 ± 1.88	6.14
**6**	18.71	609.309	525.288, 287.057	Unknown (Cy-type anthocyanin)	8.49 ± 1.34[a]	2.22
				**Total**	**382.33 ± 8.95**	**100**

Supplementary Figure 3. HPLC-DAD chromatogram achieved at 520 nm corresponding to black rice anthocyanins (1 mg/mL) after dissolving the black rice extract powder in acidified water (2% v/v formic acid). Identification of anthocyanins in black rice extract powder was achieved by freshly preparing a concentration of black rice extract powder of 1 mg/mL in 2%v/v formic acid in water and 10 μL was injected through LC-Q/TOF in positive mode. Both low and high-energy collisions were scanned alongside UV-VIS detection at 500 nm. Gradient elution was performed using 1% v/v formic acid in water as solvent A, and 1% v/v formic acid in acetonitrile as solvent B. However, the quantification method was performed by using an additional standard curve for Cy3Glc but an external standard curve for other compounds. All peaks were detected at 520 nm on HPLC-DAD using a gradient elution of 5% v/v formic acid in water as solvent A, and 5% v/v formic acid in acetonitrile as solvent B. The content was calculated using the average of three different concentrations of black rice extract powder. Values are expressed as mean ± SD.

[a] =expressed as Cy3Glc equivalent.

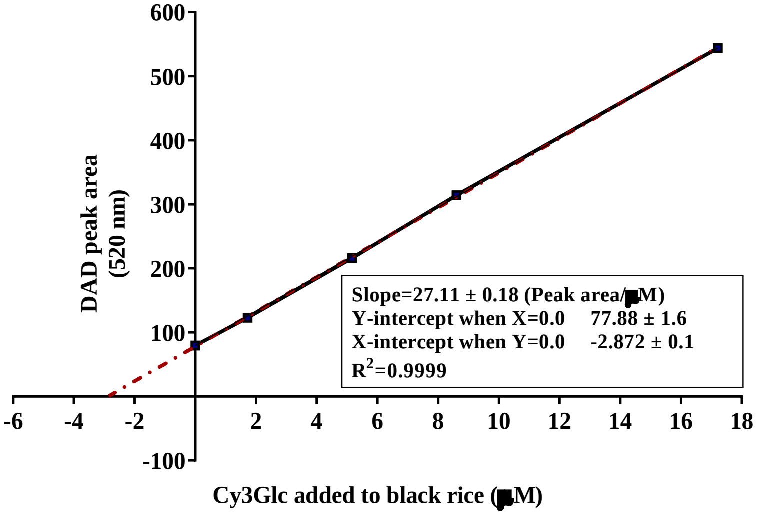

Vials	Black rice (µL)	Pure Cy3Glc (µL)	Added Cy3Glc (µM)	Acidified water (µL)	Peak area (520 nm)
**0**	0.00	0.00	0.0000	1000	0.000
**1**	250	0.00	0.0000	750	79.32
**2**	250	50.0	1.7215	700	122.8
**3**	250	150	5.1645	600	216
**4**	250	250	8.6075	500	314.2
**5**	250	500	17.215	250	543.9

Supplementary Figure 4. Quantification of black rice cyanidin-3-*O*-glucoside (Cy3Glc) using standard addition method. The stock solution of black rice extract powder (16.6667 µg/mL) prepared in acidified water (2% v/v formic acid) was mixed with either acidified water (no addition) or increasing volumes of authentic standard of 34.430 µM of Cy3Glc (in acidified water; from the same stock as used to prepare the external standard curve) and appropriate volumes of acidified water so that all samples had a final volume of 1 mL. The Cy3Glc content of the black rice extract powder was estimated by plotting the DAD (520 nm) peak area against the added concentration of pure Cy3Glc, which was 333.6 ± 15.3 mg/g dry weight powder (33.4 % w/w).

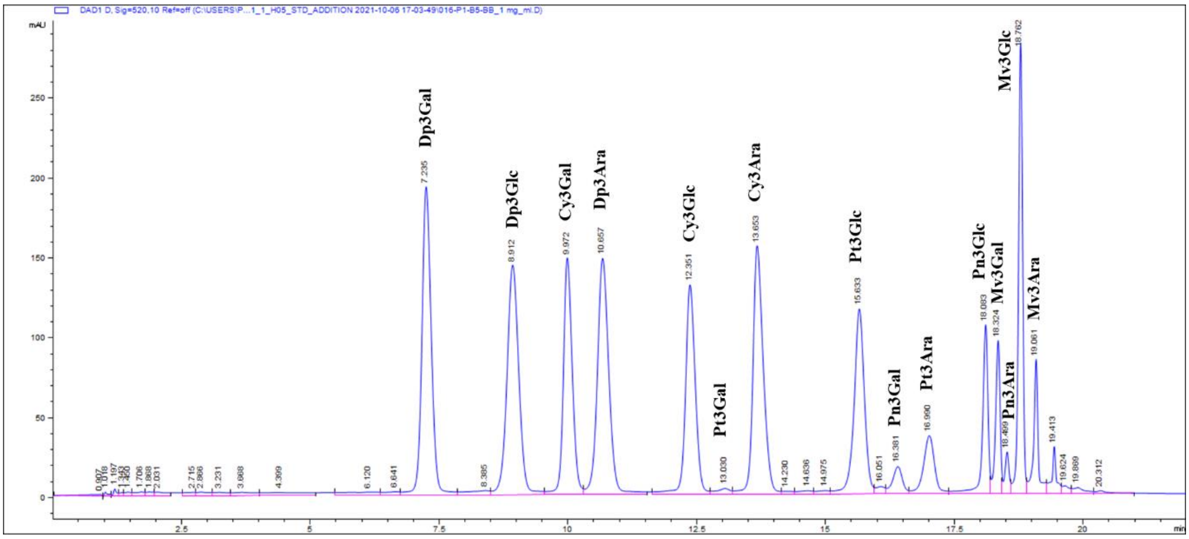

Peak	RT DAD_{520nm} (min)	Parent ion TOF-MS $[M+H]^+$	Other ions TOF-MS $[M+H]^+$	Identified anthocyanin	Content[#] (mg g^{-1} d.w.)	% of total anthocyanins
**1**	7.22	465.104	303.052	Dp3Gal	36.87 ± 0.66[a]	14.0
**2**	8.91	465.104	303.052	Dp3Glc	34.89 ± 0.67[a]	13.3
**3**	9.97	449.108	287.057	Cy3Gal	26.49 ± 0.53[b]	10.1
**4**	10.65	435.093	303.052	Dp3Ara	32.19 ± 0.68[a]	12.2
**5**	12.35	449.109	287.057	Cy3Glc	25.55 ± 0.66[b]	9.7
**6**	13.03	479.119	317.067	Pt3Gal	Trace	0.0
**7**	13.65	419.099	287.057	Cy3Ara	29.71 ± 0.61[b]	11.3
**8**	15.63	479.118	317.067	Pt3Glc	21.58 ± 0.40[c]	8.2
**9**	16.38	463.124	301.072	Pn3Gal	2.75 ± 0.12[d]	1.0
**10**	16.99	449.109	317.067	Pt3Ara	6.80 ± 0.15[c]	2.6
**11**	18.08	463.124	301.073	Pn3Glc	10.39 ± 0.18[d]	4.0
**12**	18.32	493.135	331.083	Mv3Gal	8.24 ± 0.18[e]	3.1
**13**	18.49	433.113	301.073	Pn3Ara	1.67 ± 0.11[d]	0.6
**14**	18.76	493.135	331.083	Mv3Glc	20.53 ± 0.37[e]	7.8
**15**	19.06	463.124	331.082	Mv3Ara	5.20 ± 0.12[e]	2.0
			**Total**		**262.9 ± 5.46**	**100**

Supplementary Figure 5. HPLC chromatogram achieved at 520 nm corresponding to bilberry anthocyanins (1 mg/mL) after dissolving the bilberry extract powder in acidified water (2%FA). The quantification method was performed by using an external standard curve at 520 nm on HPLC-DAD. The content was calculated using the average of five different concentrations of bilberry extract powder. Values are expressed as mean ± SD.

[a] =expressed as Dp3Glc, [b] =expressed as Cy3Glc, [c] =expressed as Pt3Glc, [d] =expressed as Pn3Glc, and [e] =expressed as Mv3Glc equivalents.

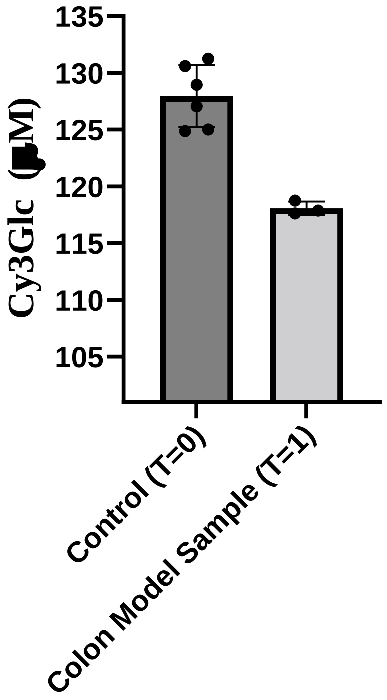

Supplementary Figure 6. Initial rapid loss of Cy3Glc in the colon model. Black rice extract (18 mg powder, containing 33.36 % w/w Cy3Glc) was dissolved in 1 mL water, filtered and immediately added to a colon model vessel pre-filled with sterile media (89 mL) and human faecal slurry (10 mL of a 10% slurry from a fresh stool) to give a final volume of 100 mL and a Cy3Glc concentration of 133.60 µM (60 µg/mL). Colon model samples (0.5 mL, *n*=3) were collected within 1 min after adding the black rice extract, mixed with 0.5 mL of 4 % v/v aqueous formic acid, and after sample preparation, analysed using HPLC-DAD to determine the Cy3Glc concentration. Control sample (0.5 mL, *n*=5) were prepared by spiking filtered black rice extract directly to a vial contains 50% FA water and 50% inoculated media (10 % faecal slurry). The concentration of Cy3Glc in colon model samples were significantly different from the concentration of Cy3Glc in control samples (T=0).

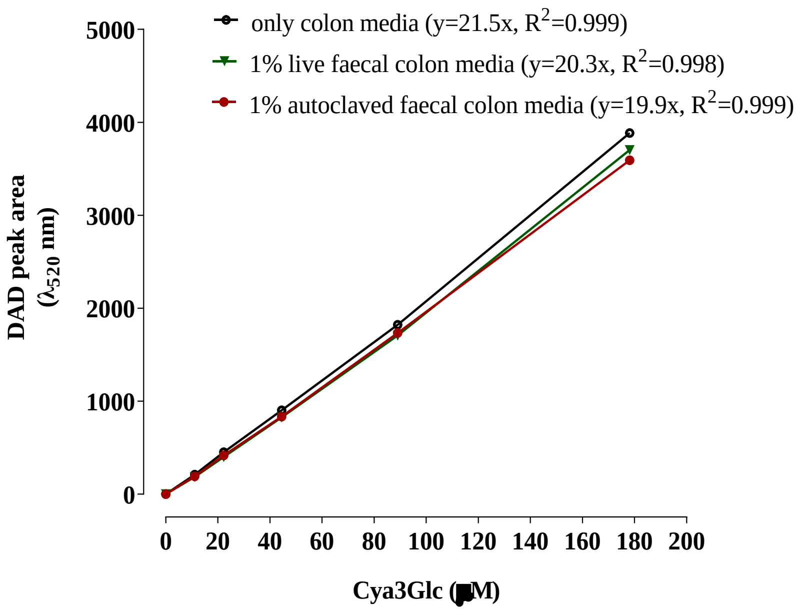

Supplementary Figure 7. The effect of different matrices on the slope of a standard curve of the black rice Cy3Glc. Stool sample from donor C-S3 was collected, and three different matrices were prepared as following: (i) colon media with 0% faecal sample, (ii) 1% live faecal sample, and (iii) 1% autoclaved faecal sample. Stock Solution of Cy3Glc (500 µg/mL) was prepared in 5 mL colon media (pH 7) by dissolving 9 mg black rice extract. Serial concentrations (0 (0), 5 (11.1), 10 (22.5), 20 (44.5), 40 (89.1), and 80 µg/mL (178.2 µM)) were immediately prepared separately in the three matrices including 50% v/v acidified water (4%FA v/v). The prepared samples were vortexed followed by centrifugation for 10 min (17000 x g, at 4 ºC). The supernatant was injected onto HPCL-DAD and Cy3Glc was detected at 520 nm.

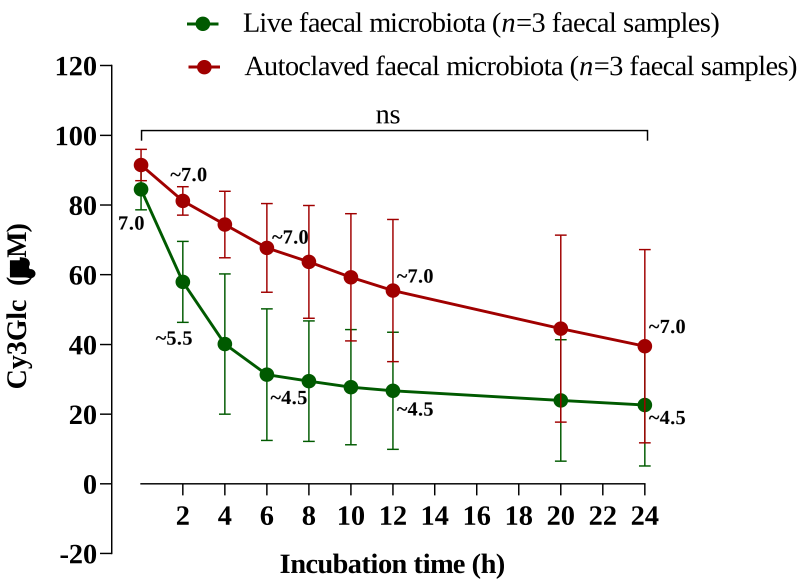

Supplementary Figure 8. The degradation of black rice Cy3Glc in a simple anaerobic cabinet stirred vessels human colon model that is not pH controlled. Black rice extract (9 mg, containing 33.36 % w/w Cy3Glc) was dissolved in 1 mL water, filtered and immediately added to a colon vessel pre-filled with sterile media (44 mL) and human faecal slurry (5 mL of a 10 % slurry from a fresh stool) to give a final volume of 50 mL and a Cy3Glc concentration of 133.60 µM (60 µg/mL). Control vessels were prepared by incubating fresh faecal inoculum in the same media but without black rice extract. Control vessels were prepared by incubating autoclaved faecal samples. Incubation was carried out anaerobically at 37°C, over 24 h. Samples (0.5 mL) were collected at the times shown in the figure, mixed with 0.5 mL of 4 % v/v aqueous formic acid, and after sample preparation, analysed using HPLC-DAD to determine the Cy3Glc concentration. No Cy3Glc was detected in control vessels lacking black rice extract (data not shown). The data shown are for 3 replicate incubations for each condition using a single donor faecal sample: donor A-S7 (*n*=1), donor E-S1 (*n*=1), and donor E-S2 (*n*=1). Values represent means ± SD. Statistical analysis was carried out with one-way ANOVA with Tukey multiple comparisons for each time point.

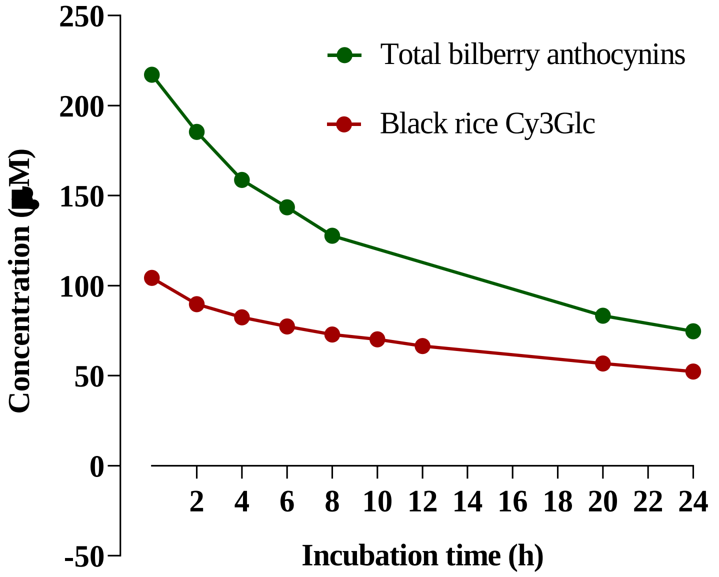

Supplementary Figure 9. Changes in concentrations of black rice Cy3Glc and total bilberry anthocyanins over time in colon model media in the absence of faecal slurry. In the absence of any faecal materials, the $k_{deg(0\text{-}2\ h)}$ between different bilberry anthocyanins showed constant rates over the different time points. This contrasts with the anthocyanins incubated with the autoclaved sample which showed relatively higher k_{deg} in the first 2 h compared to 4, 6, and 8 h time points. This suggests that faecal material affects the k_{deg} of anthocyanins.
